# Supplementary material for: Statistical crystallography reveals an allosteric network in SARS-CoV-2 Mpro
Source: Commun Biol. 2026 May 2;9:602. doi: 10.1038/s42003-026-10127-w (PMC13135515; doi:10.1038/s42003-026-10127-w)
Supplement: Supplementary file 1 — Supplementary Information [file 42003_2026_10127_MOESM1_ESM.pdf]

# Supplementary Materials for

## Statistical crystallography reveals an allosteric network in SARS-CoV-2 $M^{pro}$

Creon *et al.*

*Corresponding author. Email: thomas.lane@desy.de

**This PDF file includes:**

Figs. S1 to S16
Tables S1 to S4
Supplemental References

**Other Supplementary Materials for this manuscript include the following:**

Data S1 to S5

S1. SEC raw data
S2. SEC fit data
S3. ITC and nDSF raw data
S4. Raw reaction velocities
S5. Fit Michaelis-Menten parameters

| dataset | cell volume ($nm^3$) | ensemble size | resolution $CC_{1/2} > 0.5$ | single $R_{work}$ | single $R_{free}$ | ensemble $R_{work}$ | ensemble $R_{free}$ |
|---|---|---|---|---|---|---|---|
| **A** | 256.28 | 67 | 1.98 | 0.1997 | 0.2378 | 0.1566 | 0.2126 |
| **B** | 260.66 | 50 | 1.65 | 0.1791 | 0.2058 | 0.1577 | 0.2075 |
| **C** | 264.53 | 29 | 1.77 | 0.1955 | 0.2342 | 0.1693 | 0.2250 |
| **D** | 270.65 | 50 | 1.72 | 0.1870 | 0.2173 | 0.1627 | 0.2174 |

**Supplemental Table 1. Ensemble refinement statistics**. Ensemble refinements were performed on datasets selected to span the observed range of cell volumes to increase the diversity of the data analyzed. Reported are the number of conformations that *phenix* automatically determines during the ensemble refinement procedure, as well as *R*-factors for the single-conformer starting model and multi-conformer ensemble after MD-based refinement (*2*, *3*). These ensembles were used to compute covariance matrices, shown in Supplemental Fig. 6; the first column maps these datasets to the panels shown there.

| variant | [E] (µM) | $K_D$ (µM) | *ΔH* (kcal/mol) | *ΔG* (kcal/mol) | *-TΔS* (kcal/mol) |
|---|---|---|---|---|---|
| wild type | 20.7 +/- 1.2 | 2.4 +/- 0.8 | -3.9 +/- 0.3 | -7.8 | -3.9 |
| N214A | 17.5 +/- 0.6 | 2.0 +/- 0.4 | -4.4 +/- 0.2 | -7.9 | -3.5 |
| Q256A | 17.5 +/- 1.3 | 3.4 +/- 1.0 | -5.9 +/- 0.6 | -7.5 | -1.7 |
| S284A | 16.7 +/- 0.7 | 1.6 +/- 0.4 | -3.0 +/- 0.2 | -8.0 | -5.1 |

**Supplemental Table 2. Thermodynamic parameters of calpeptin binding to wild type, N214A, Q256A and S284A $M^{pro}$ derived from isothermal titration calorimetry (ITC).** Enzyme concentrations ([E]), dissociation constants ($K_D$), enthalpy changes (*ΔH*), Gibbs free energy changes (*ΔG*), and entropy contributions (*-TΔS*) for each variant:calpeptin interaction were estimated from the standard two-state model fit to ITC data (Supplemental Fig. 9). Fits performed with MicroCal PEAQ-ITC. The comparable $K_D$ values suggest similar ligand binding affinities across the different mutants. Errors are standard errors of the mean, as reported by MicroCal PEAQ-ITC v1.41.

| **variant** | **[E] (μM)** | ***n*** | **$K_D$ (μM)** | **fit method** |
|---|---|---|---|---|
| wild type | **2** | **3** | **0.5 +/- 0.2** | **averaging** |
| | 10 | 3 | 0.4 +/- 0.2 | averaging |
| | combined | 6 | 0.43 +/- 0.12 | least squares |
| N214A | **2** | **3** | **5.6 +/- 0.9** | **averaging** |
| | 10 | 3 | 6 +/- 3 | averaging |
| | 20 | 3 | 4 +/- 3 | averaging |
| | combined | 9 | 5.9 +/- 1.4 | least squares |
| Q256A | **2** | **3** | **0.80 +/- 0.11** | **averaging** |
| | combined | 3 | 0.80 +/- 0.14 | least squares |
| S284A | **2** | **3** | **0.56 +/- 0.11** | **averaging** |
| | combined | 3 | 0.57 +/- 0.13 | least squares |

**Supplemental Table 3. Dimerization constants determined by native mass spectrometry.** To investigate the possibility of systematic errors as a function of concentration, monomer/dimer $K_D$s were computed from native mass spectrometry data one concentration at a time via simple averaging, and in aggregate by fitting the standard two-state law of mass action with non-linear least squares. Within error, the determined affinities are consistent. As low concentration measurements are expected to suffer the least systematic error, affinities determined at 2 μM (bold) were reported in the main text and employed in subsequent analysis. All uncertainties are standard errors assuming a Gaussian error model.

| model | (ii) third order | | (iii) dimer pre-equilibrium | | |
|---|---|---|---|---|---|
| **variant** | $k_{cat}$ (min$^{-1}$) | $K_{eq}$ (μM$^2$) | $k_{cat}$ (min$^{-1}$) | $K_M$ (μM) | $K_D$ (μM) |
| wild type | 1.6 +/- 0.4 | 1000 +/- 700 | 0.88 +/- 0.2 | 107 +/- 3 | 0.30 +/- 0.02 |
| N214A | 0.11 +/- 0.06 | 10 000 +/- 13 000 | 0.100 +/- 0.005 | 220 +/- 10 | 4e-26 +/- 8e-6 |
| Q256A | 0.8 +/- 0.2 | 900 +/- 500 | 0.32 +/- 0.02 | 54 +/- 3 | 0.18 +/- 0.04 |
| S284A | 2.1 +/- 0.6 | 2000 +/- 1000 | 0.50 +/- 0.01 | 57 +/- 2 | 0.019 +/- 0.005 |

**Supplemental Table 4 Fit parameters for alternative kinetics models.** See *Materials and Methods* for model details and Supplementary Fig. 11 for a visualization of the data and fits. All errors are 95% confidence intervals.

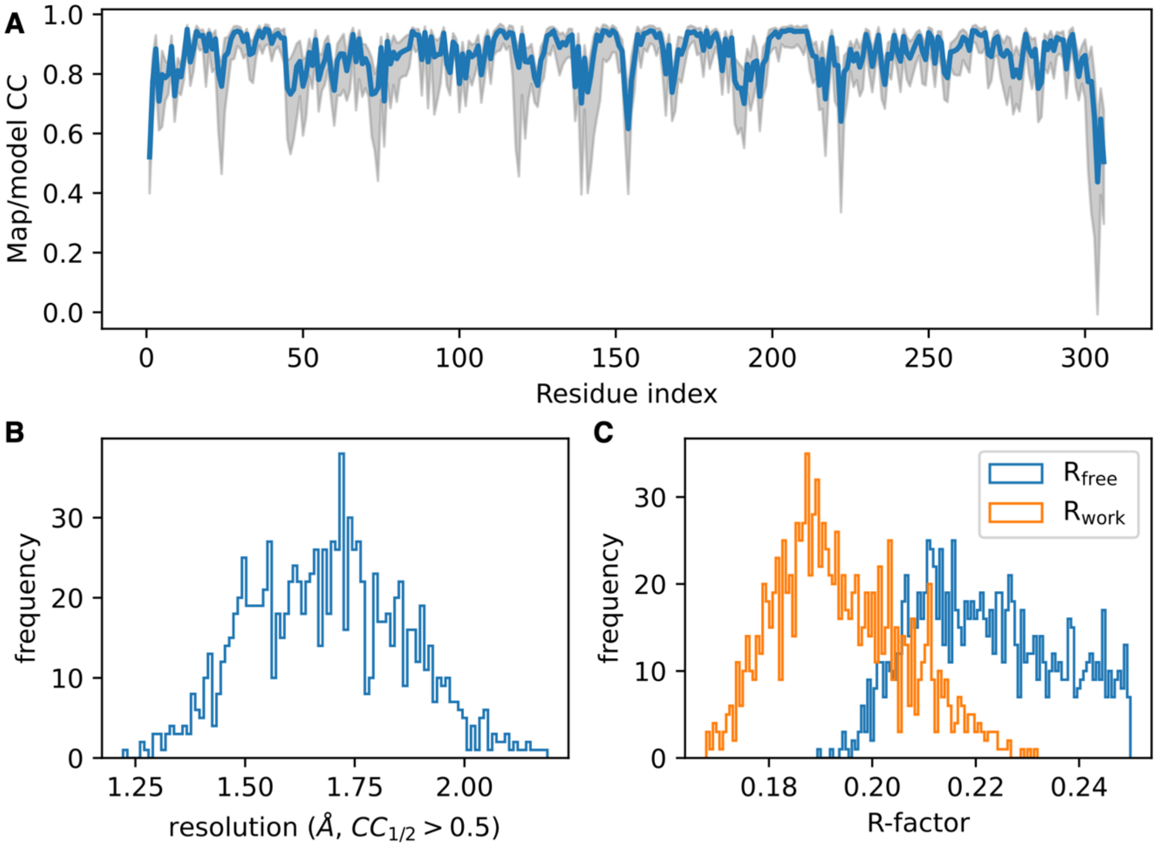


**Supplemental Figure 1. Overview of the data quality of the 1146 structure dataset.** (**A**) Real-space correlation between the $2mF_o$-$DF_c$ map and the model ($F_c$) for the set of structures presented as a function of residue (all atoms in a residue averaged). The mean value is shown in blue, while the extrema across all structures shown in grey. (**B**) Histograms showing the distribution of dataset resolution limits, determined as where $CC_{1/2}$ falls below 0.5, and (**C**) refinement R-factors.

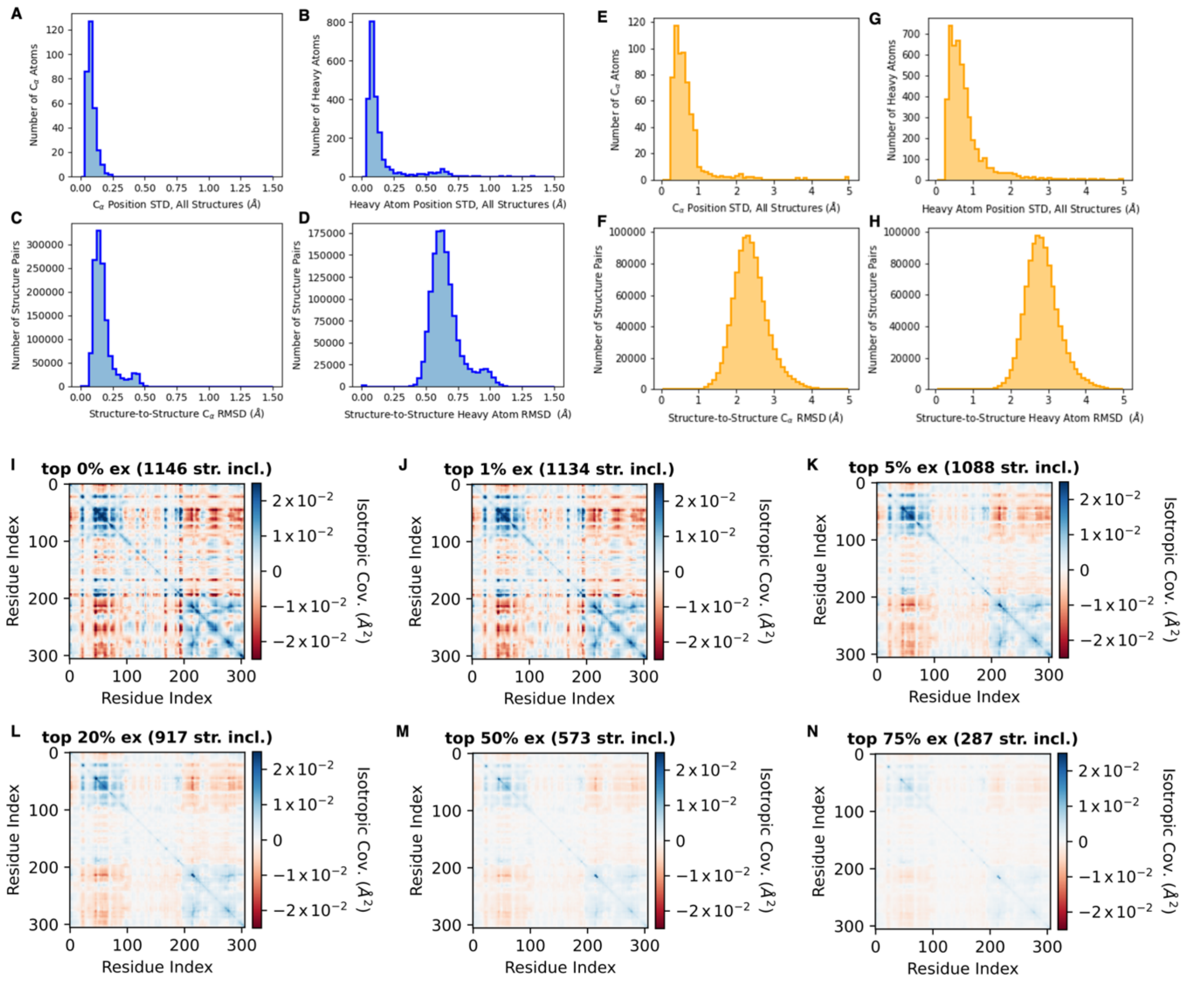


**Supplemental Figure 2. Variability of the 1146 structure dataset.** (**A**, **B**) The standard deviation of the refined atom positions provides a measure of the structural variability in the dataset produced by the automatic refinement procedure. Shown are (**A**) $C_\alpha$ atoms only and (**B**) all heavy atoms. Similarly, (**C**, **D**) the distribution of all pairwise structure RMSD values, again for (**C**) $C_\alpha$ atoms only and (**D**) all heavy atoms. The structural distribution is narrow, but non-trivial; clear bimodal behavior, for example, is apparent in this simple representation. (**E**-**H**) The same plots, for the DESRES MD simulation, note the change in x-axis range. The MD simulation samples a significantly larger set of conformational space. (**I**-**N**) While many of the crystal structures are similar, with a low RMSD spread, a substantially sized minority deviate more strongly from the mean and disproportionately drive the covariance signal observed. Plots (**I**-**N**) present a data ablation study, in which the most distinct structures – as measured by the mean of the $C_\alpha$ RMSD from that structure to all other structures in the set – were systematically excluded from analysis. Covariance matrices were then computed from the remaining data: (**I**) no data excluded, (**J**) the 1% most distinct structures excluded, (**K**) similarly but 5% excluded, (**L**) 20%, (**M**) 50%, (**N**) 75%. The number of remaining structures is indicated above the corresponding plot, abbreviated "str. incl.". Excluding the most distinct 5% of structures, the rare excursions from the center of the distribution, significantly attenuates the covariance signal.

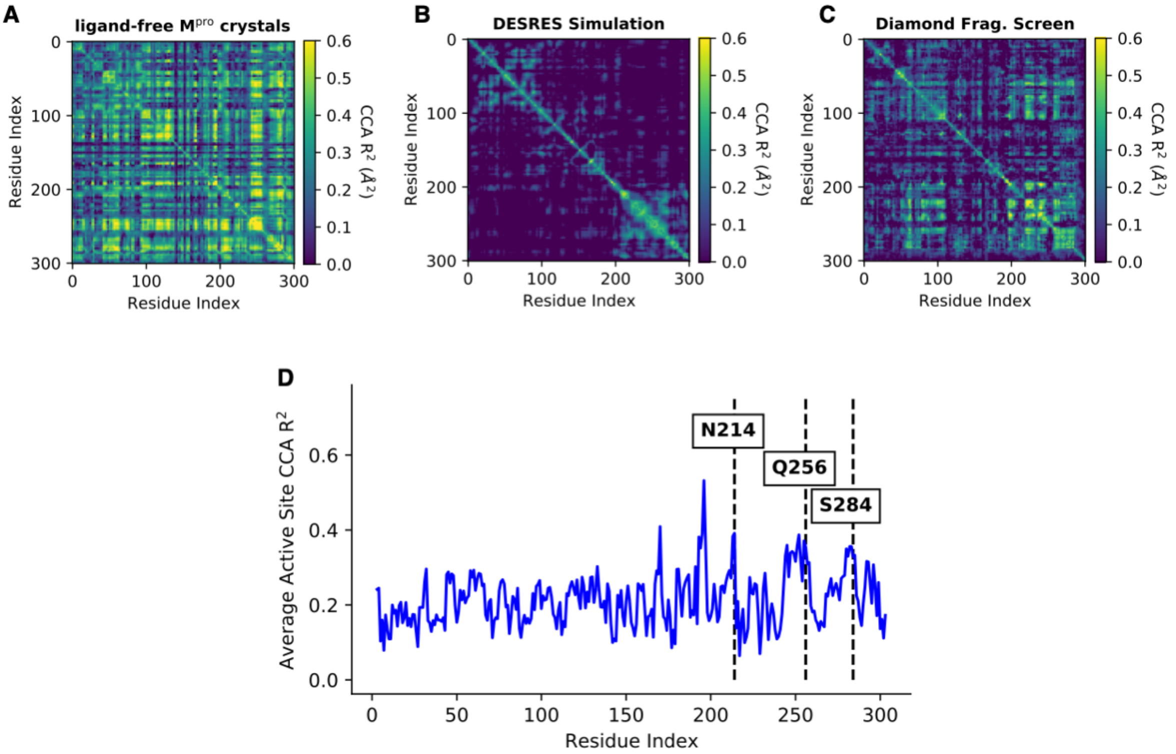


**Supplemental Figure 3. Canonical correlation analysis of $M^{pro}$.** Given a pair of atoms (here $C_{\alpha}$), the canonical correlation reports the maximum correlation between any two linear functions of the Cartesian coefficients of each atom (x, y, z) (*4*). This differs from the covariance presented in the main text in three key ways. First, it accounts for situations where atomic correlations are non-isotropic, for example should the *x*-coordinate of atom *i* influence the *y*-coordinate of atom *j*. Second, it computes a correlation rather than a covariance, and therefore normalizes for the total magnitude of the atomic displacement. Third, because rotations and translations are linear functions, the analysis is equivariant under such operations. For instance, for $M^{pro}$ in the C2 space group when comparing an intra-protomer atom pair to a cross-dimer atom pair, the direction (but not the magnitude) of the correlation/covariance may change, but the canonical correlation will be invariant. Reported here are the coefficients of determination ($R^2$) for the atom-atom canonical correlation models of (**A**) our set of 1146 crystal structures, (**B**) the DESRES MD simulation, and (**C**) the Diamond $M^{pro}$ structures, as presented in main text Figure 2. Further, shown is (**D**) the coefficients of determination averaged over active site residues, analogous to main text Figure 3C.

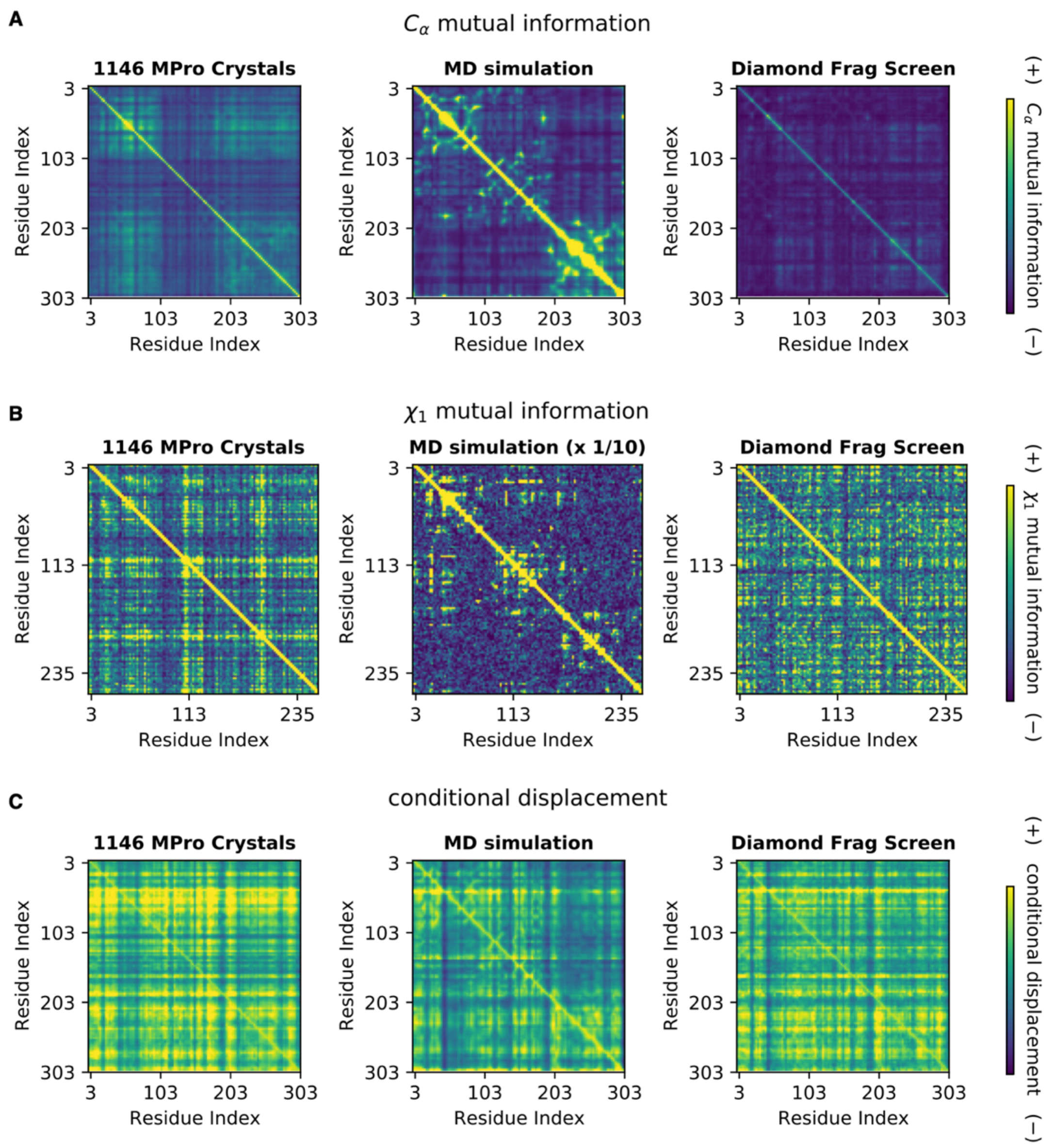


**Supplemental Figure 4. Alternative measures of correlation.** Reported are (**A**) the mutual information between $C_\alpha$ cartesian positions, (**B**) the mutual information between sidechain $\chi_1$ dihedral angles, and (**C**) the conditional displacement of $C_\alpha$ atoms (see *Methods and Materials* for definitions). Mutual information is able to capture non-Gaussian behavior, but requires more samples to converge than the covariance measure employed in the main text. The conditional displacement assumes Gaussian displacements, but can capture anisotropic displacements, and therefore also requires additional data to converge. The MD simulation (middle column), consisting of 10,000 structures sampled across 100 μs of simulation time, shows the structure of the correlations present in the protein in the mutual information metrics (top two rows). In contrast, for the crystalline set (rightmost and leftmost columns), the 1146 and 95 crystal structure, respectively, exhibit poorly converged digitized structure for all three measures reported in this figure. Note the MD simulation color scale zoomed out by a factor of 10 in the $\chi_1$ mutual information plot (central panel in B).

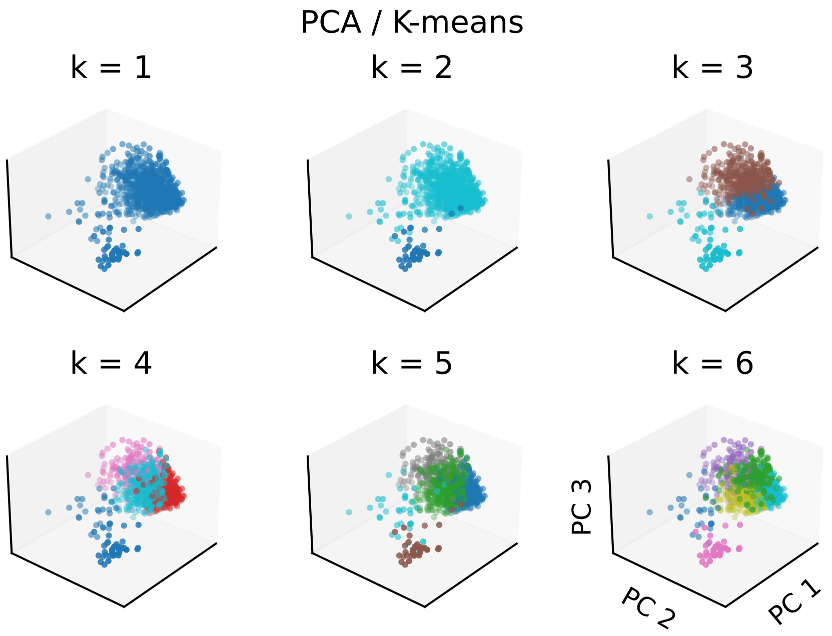


**Supplemental Figure 5. Clustering fails to identify more than two stable groupings of M$^{pro}$ structures.** To test for clear groupings of structures, clustering was performed on the protein structures that form the crystal set in a reduced dimensionality space. To accomplish this, PCA was performed on the sine and cosine of each backbone dihedral angle of each structure, and three first (highest variance) principal components (PC1, PC2, PC3 respectively) were plotted. Accordingly, each point on the plot represents the geometry of a single structure. No clear gap in the PCA spectra (eigenvalues) was observed, and therefore the first 10 (highest variance) principal components (48% of the total variance) were subsequently clustered using *k*-means (*5*), with the number of clusters (*k*) ranging from 1 to 6 as indicated above each sub-plot. The resulting cluster labels were used to color the points in each plot. Two stable clusters can be readily identified, but beyond that, the data form a continuous manifold. Therefore, we elected to not proceed further with a clustering analysis but instead focus on covariance as a way to encapsulate and analyze the structural variability of the set of crystal structures.

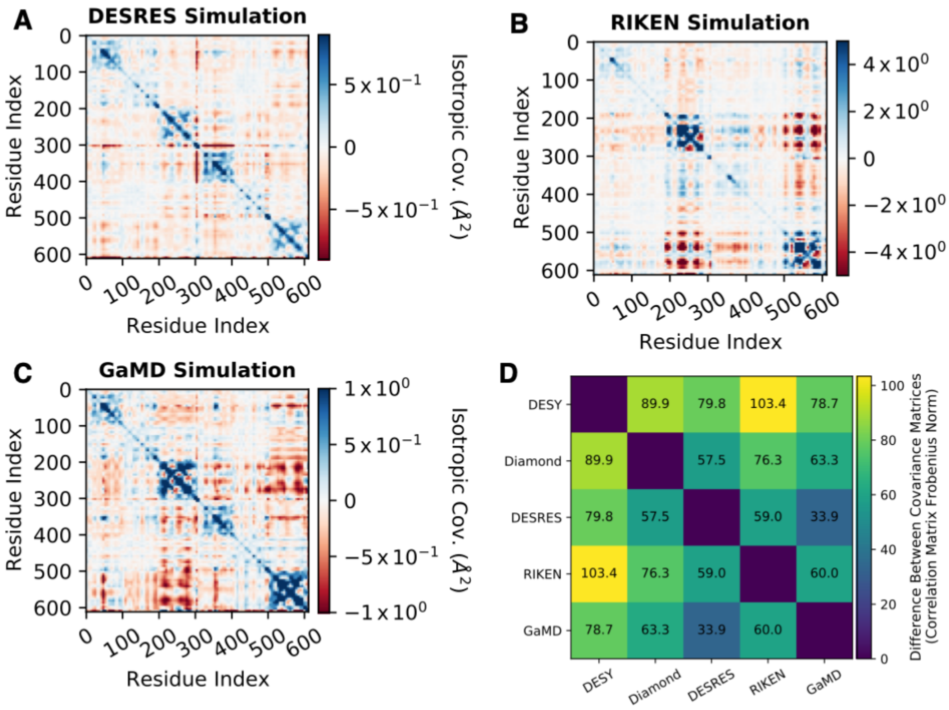


**Supplemental Figure 6. Comparison to other MD simulations.** Shown are covariance predictions for three distinct simulations of the wild-type M^pro dimer, (**A**) the 100 μs DESRES trajectory (*6*), presented in the main text (**B**) two 10 μs trajectories made available by RIKEN (*7*), and (**C**) 5 distinct 200 ns Gaussian accelerated simulations reported by Sztain *et al.* (GaMD) (*8*). All simulations were subsampled at 1 ns snapshot intervals. Panel (**D**) shows a quantitative heuristic of similarity between these covariance matrices and those from the crystallographic distributions ("DESY" corresponding to this work). Lower numbers and darker colors indicate more similar covariance matrices. Specifically, the pixel colors and numerical values report the Frobenius norm between the correlation-normalized covariance matrices. The comparison was only performed over a single monomer to facilitate comparison to the crystal structures, specifically residues 1-303 inclusive, as not all the Diamond crystals structures have the entire C-terminus modeled. The covariance models are generally in agreement, with the closest resemblance between the DESRES and GaMD simulations, followed by the crystallographic datasets, with the RIKEN simulation being the most distinct. Note that in addition to sampling, differences between the MD simulations may be due to the simulation configuration, with (A) the DESRES simulation based on the 6Y84 crystal structure, performed at 298 K with the DES-Amber force field, with His80 protonated at Nδ and other histidines protonated at Nε. In contrast, (B) the RIKEN simulation was started from the 6LU7 crystal structure, run at 310 K using Amber99sb-ildn, with His64 and His80 protonated at Nδ and other histidines at Nε and (C) the GaMD simulations used the 6LU7 crystal structure, 310 K, Amber ff14SB force field, with His64, His80 and His164 protonated at Nδ and all other histidines protonated at Nε.

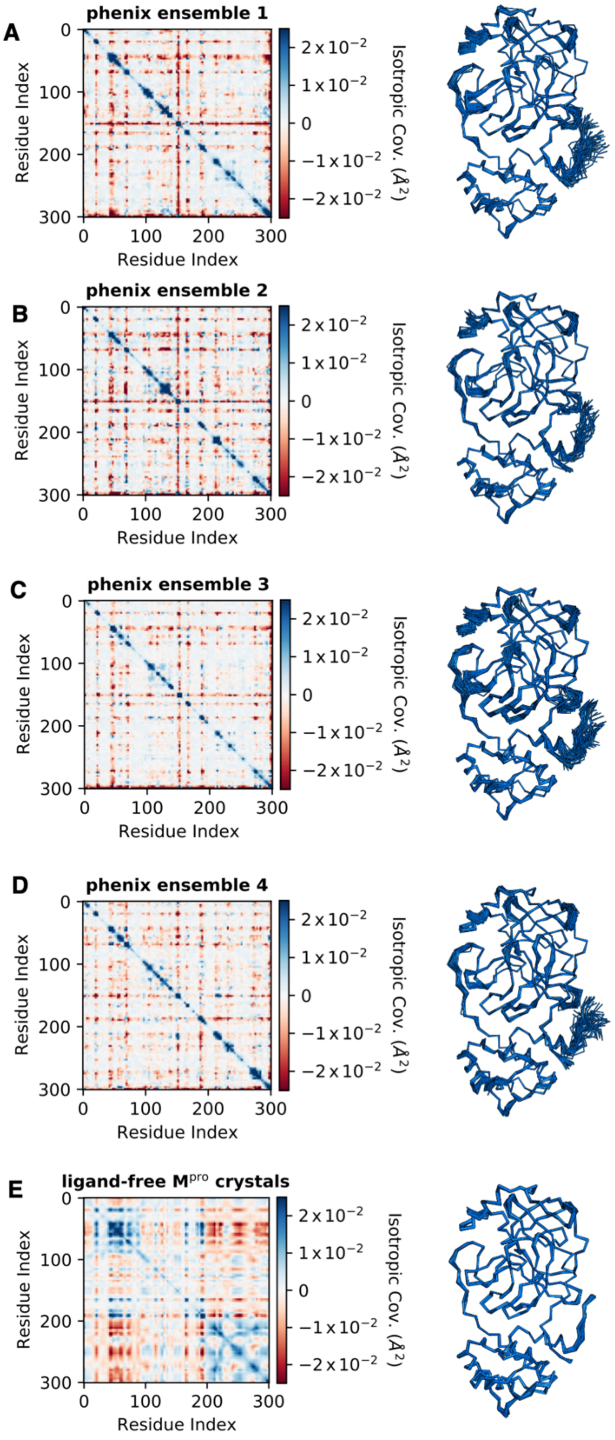


**Supplemental Figure 7. Ensembles fit to single crystal datasets do not reproduce the covariance structure provided by multiple crystalline datasets.** Because our automatic refinement procedure employed MD-driven simulated annealing and used standard crystallographic restraints that are effectively an MD forcefield, we tested the idea that the distribution of structures we observed in our crystallographic ensemble might simply be the result of the forcefield prior used during refinement, and not the experimental data themselves. We conducted ensemble refinement with *phenix* (*2*, *3*) on four datasets chosen to span the observed cell volumes, a structurally diverse and representative sample from our collection of $M^{pro}$ crystal data. (**A**-**D**) The resulting ensembles and covariance matrices are qualitatively different from the ensemble formed by (**E**) the collection of all crystal structures. See Table S2 for cell volumes, ensemble size, and *R*-factors for these four datasets.

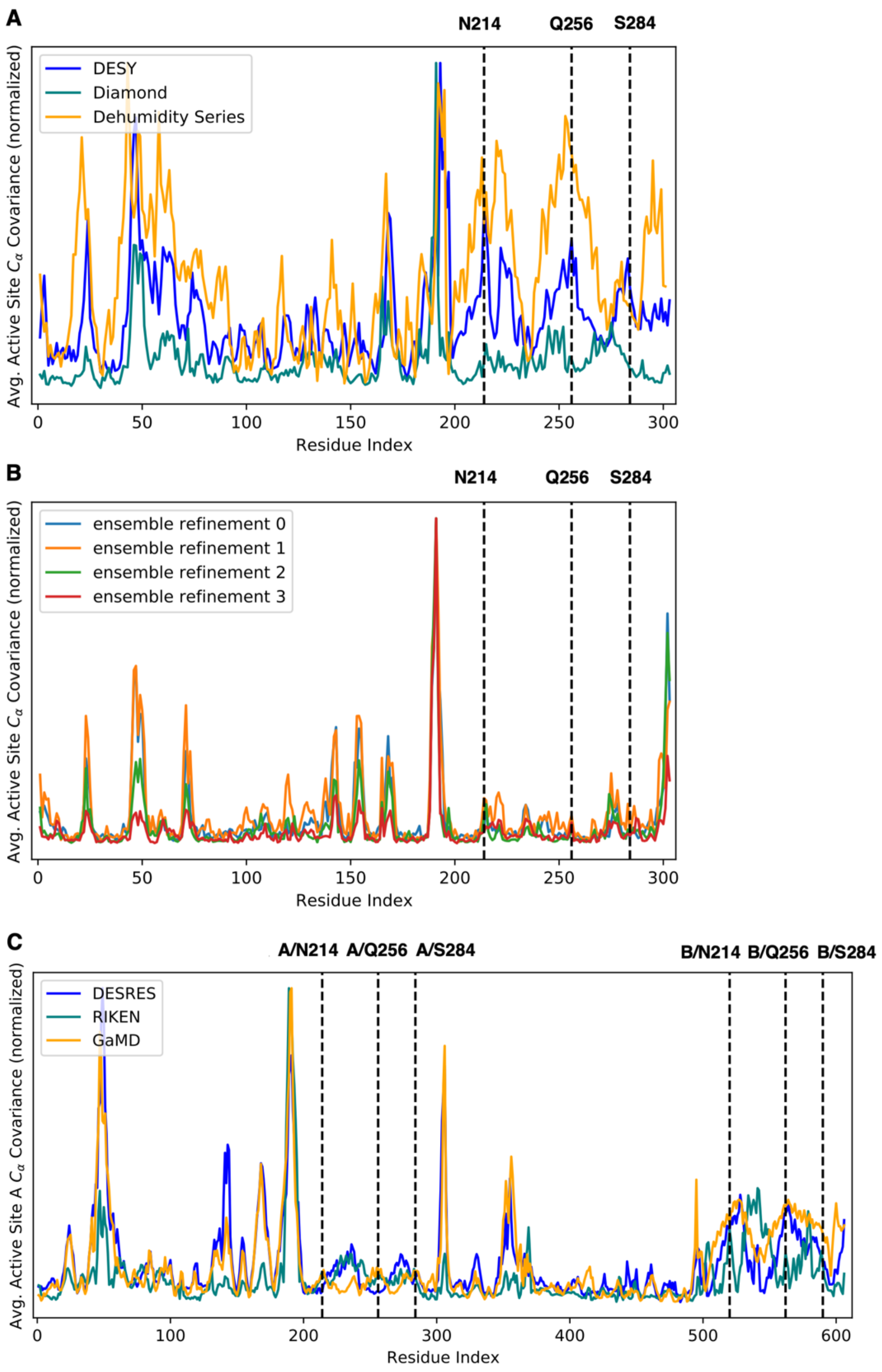


**Supplemental Figure 8. Active site** $C_\alpha$ **covariance for distinct sets of structures,** as show in main text Figure 3. (**A**) The set of 1146 structures presented in the main text (labeled DESY), the set of 95 structures from Diamond (*9*), and the 7 structures from a humidity series, where crystals were fished at fixed time intervals after opening a crystallization well and exposing the contained crystals to dry air (see main text *Materials and Methods* and Supplemental Fig. 14). (**B**) The same, for the four ensemble refinements (see Supplemental Table 1 and Supplemental Fig. 6). (**C**) For the three analyzed MD simulations, originating from DESRES (*6*), RIKEN (*7*), and Sztain *et al.* (GaMD) (*8*). For the MD simulations, the contributions of the two distinct protomers are not averaged by crystallographic symmetry; presented is the covariance of both protomers to a single active site (arbitrarily labeled "A", with the other protomer labeled "B").

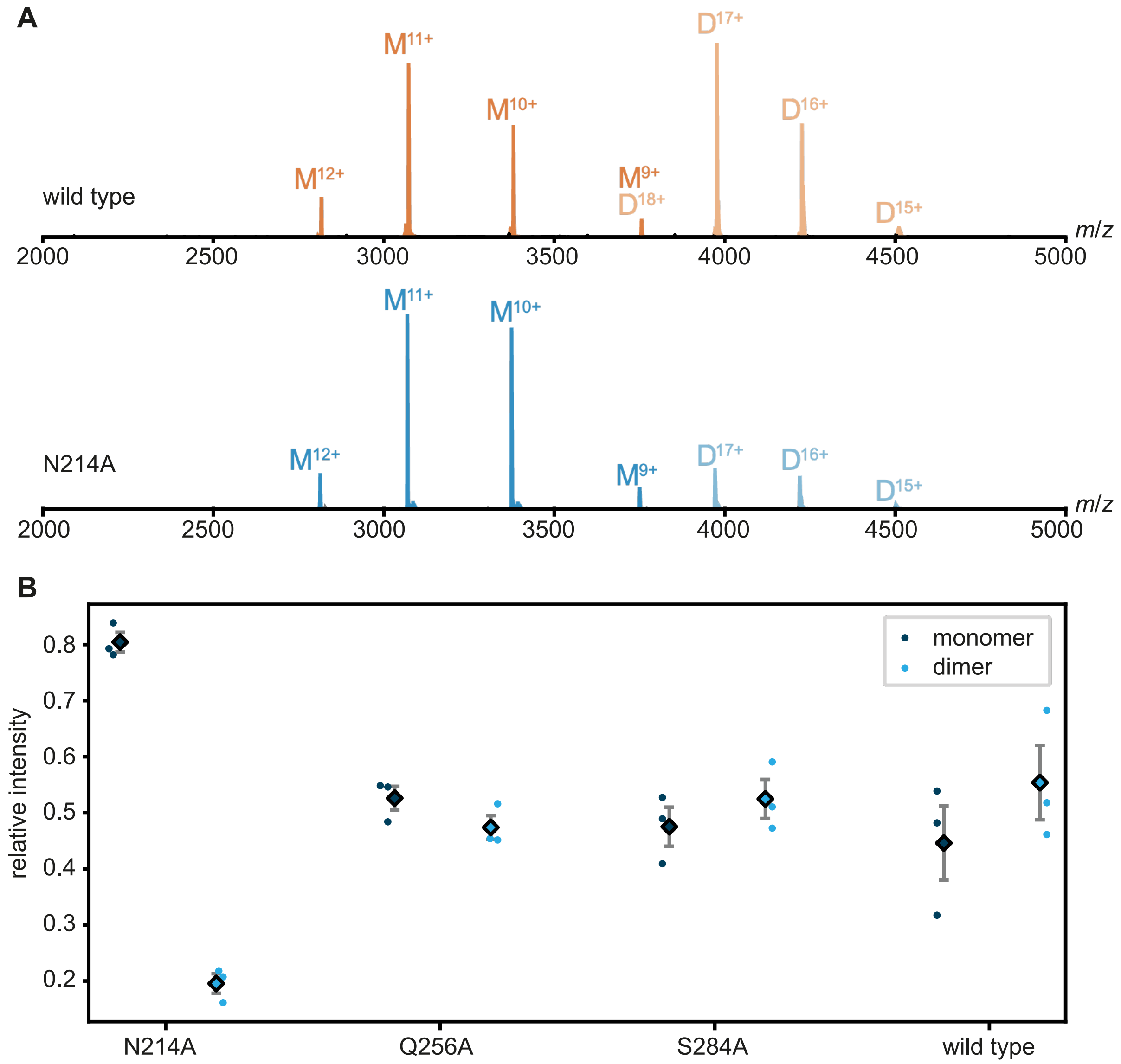


**Supplemental Figure 9. Native MS shows decreased dimer/monomer ratio for N214A variant as compared to wild type and other mutants.** (**A**) Representative native mass spectra of 2 µM wild type (top) and 2 µM N214A (bottom) showing distinct species distribution. N214A exhibits predominantly monomer peaks, whereas monomer and dimer are equally distributed in wild type. (**B**) Based on triplicate measurements, the bar chart shows the average relative intensities, proportional to the species concentration of monomers and dimers of all variants at 2 µM (see Supplemental Table 3). Error bars are standard errors.

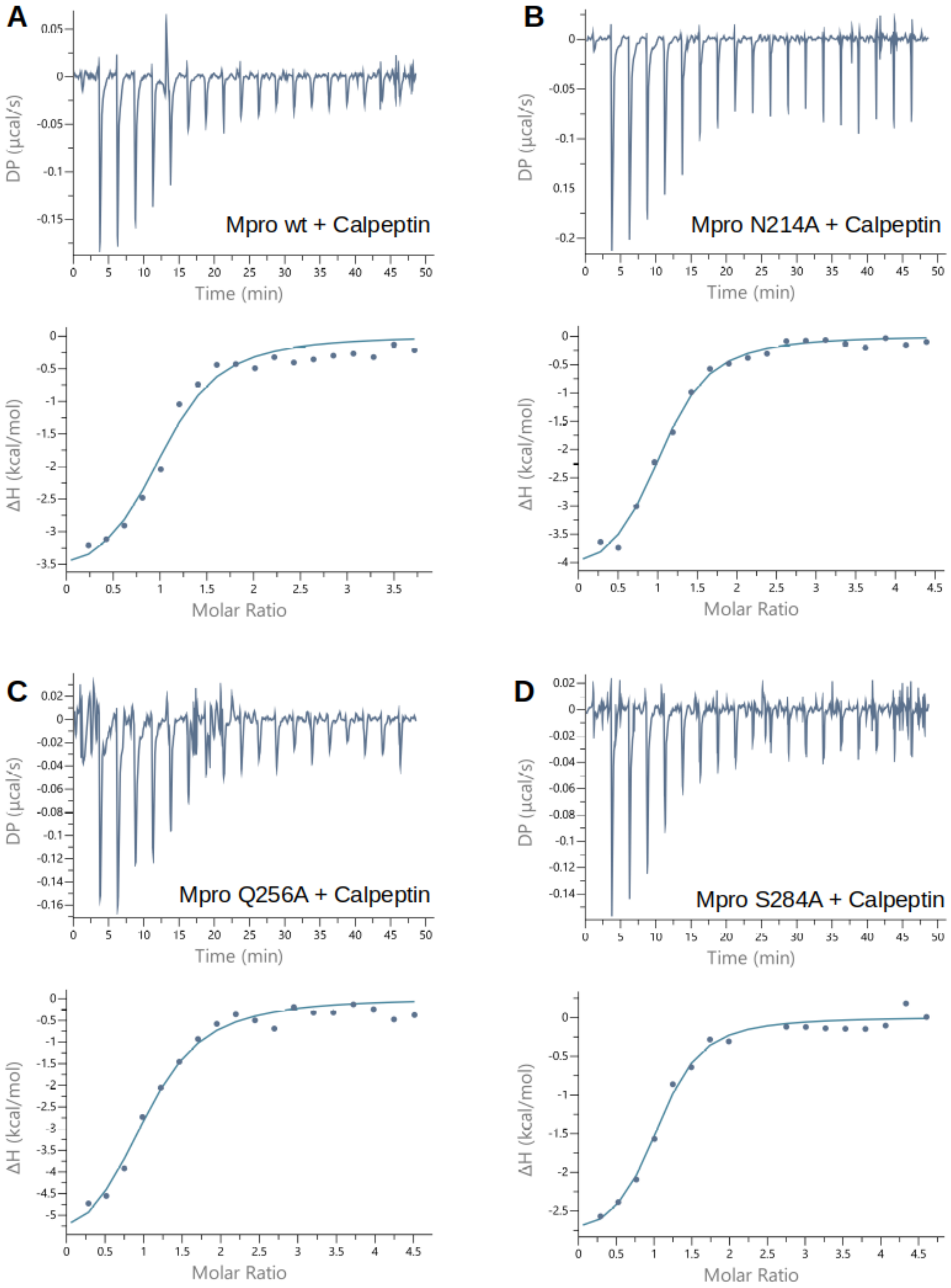


**Supplemental Figure 10. Isothermal titration calorimetry (ITC) shows ligand affinity is similar for all $M^{pro}$ variants studied.** Shown are the binding isotherm and fit for (**A**) wild type, (**B**) N214A, (**C**) Q256A and (**D**) S284A. Upper panels show the instrument power during the course of ligand injections. Lower panels show the evolved enthalpy per mol of injectant (calpeptin) against the ligand/protein molar ratio. Two points in the lower panel of (D) were rejected as outliers by the ITC software during the data analysis stage. The similarity of all binding isotherms and the ligand $K_D$ from Table 3 indicates a minor effect of the mutations studied on the ligand binding affinity. See Supplemental Table 2 for thermodynamic parameters obtain from isotherm fits.

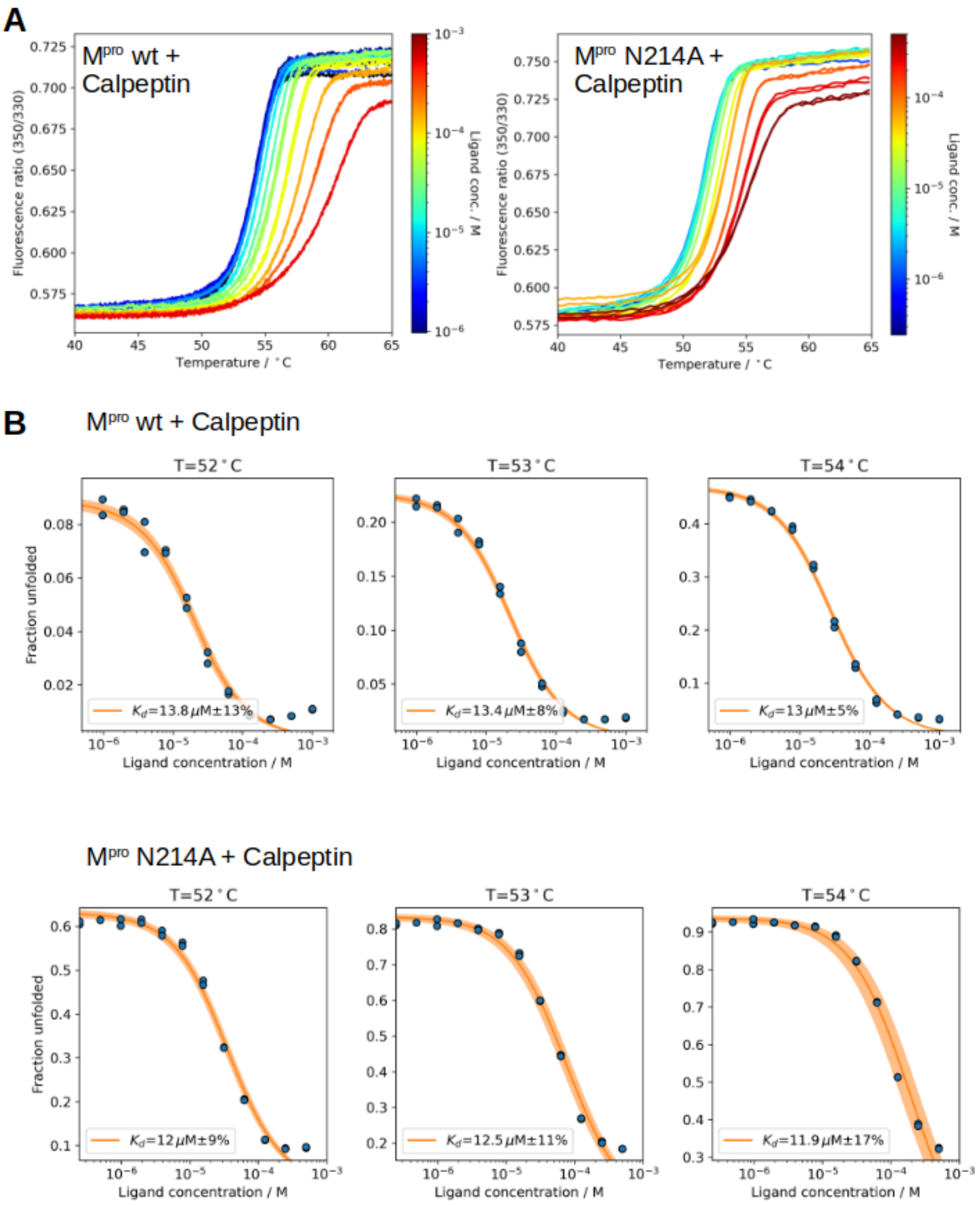


**Supplemental Figure 11. Nano differential scanning fluorimetry (nDSF) confirms ITC-derived binding affinity of M$^{pro}$ N214A.** To confirm the affinity of calpeptin for M$^{pro}$ N214A (ligand $K_D$), we conducted nDSF experiments. (**A**) Fluorescence ratio 350 nm/330 nm for nDSF binding studies of wild-type M$^{pro}$ and M$^{pro}$ N214A in the presence of increasing concentrations of calpeptin. **B**) Isothermal analysis for three selected temperatures, each showing the fraction of unfolded protein, determined by changes in intrinsic fluorescence (emission ratio at 350 nm/emission at 330 nm), as a function of ligand concentration. Fit dissociation constants (ligand $K_D$) determined from the curve fits are approximately 13 µM for the wild type and 10 µM for the N214A variant. The two tested proteins show slightly different melting temperatures of 54 °C (wild type) and 51 °C (N214A). Isothermal analysis can estimate the $K_D$ close to the melting temperature of the protein (*10*). Note the ligand affinity is reduced by about a factor of five in our nDSF measurement as compared $K_D$s determined from ITC (Supplemental Table 2); the higher temperature of the nDSF experiment is the most likely origin of this discrepancy, though we did not investigate this further. Nevertheless, the wild-type M$^{pro}$ and the N214A mutant ligand affinities are not distinguishable within the error of the nDSF measurement, demonstrating the N214A mutant's ligand binding capabilities are not greatly compromised. All plots shown here were generated with BioPhyPy (*11*).

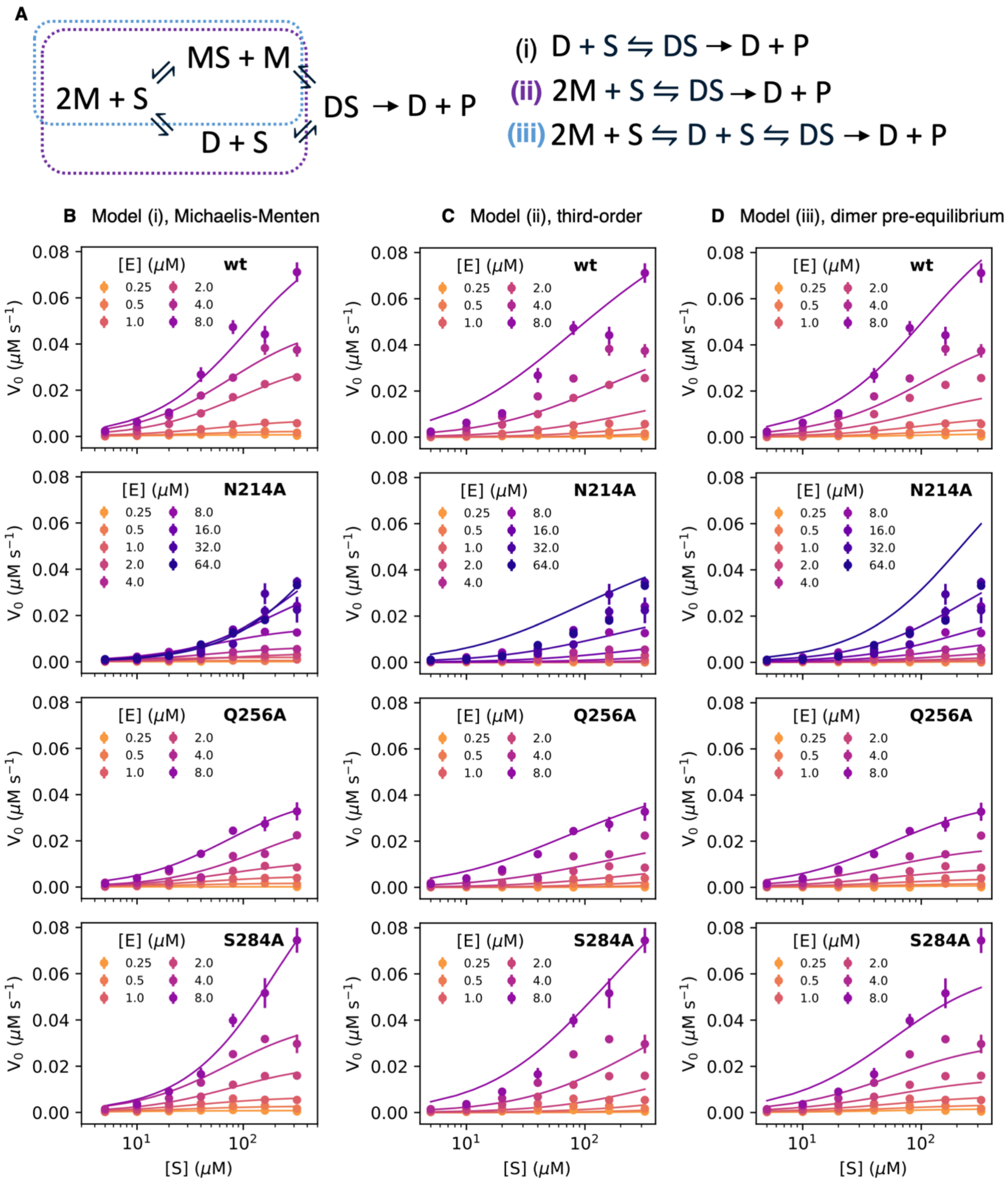


**Supplemental Figure 12. Enzyme kinetics models considered.** (**A**) Elaborated kinetic scheme for catalysis by $M^{pro}$ dimers. As this full scheme contains many fundamental rates that cannot be unambiguously determined, we assessed simplified models to explain observed initial reaction velocities $V_0$ as a function of total enzyme concentration [E] and substrate concentration [S]. Additional details and equations can be found in the *Materials and Methods.* (**B**) In model (i), each enzyme concentration is treated as an independent Michaelis-Menten scheme, as presented in the main text (Fig. 4). (**C**) In model (ii), we assume substrate equilibrates rapidly with both monomer and dimer species, which in turn equilibrate rapidly with one another (species inside purple box in (A)), leading to third-order association kinetics with a fit $K_{eq}$. This model provides a slightly worse fit, with a comparable interpretation from the fit parameters (Supplemental Table 4). Note that N214A is not well described by this model and the kinetic parameters are meaningless. (**D**) Finally, in model (iii), the monomer/dimer equilibration is assumed to be slow with respect to the rate of formation of a dimer-substrate complex, such that for each enzyme concentration a fixed concentration of dimers turns over substrate with Michaelis-Menten kinetics (species in blue box in (A)). This model fits the observed data reasonably well for all variants except N214A. While qualitatively different, the qualitative conclusions drawn from each model are consistent. See Supplemental Table 4 for fit parameters. Error bars are 95% confidence intervals.

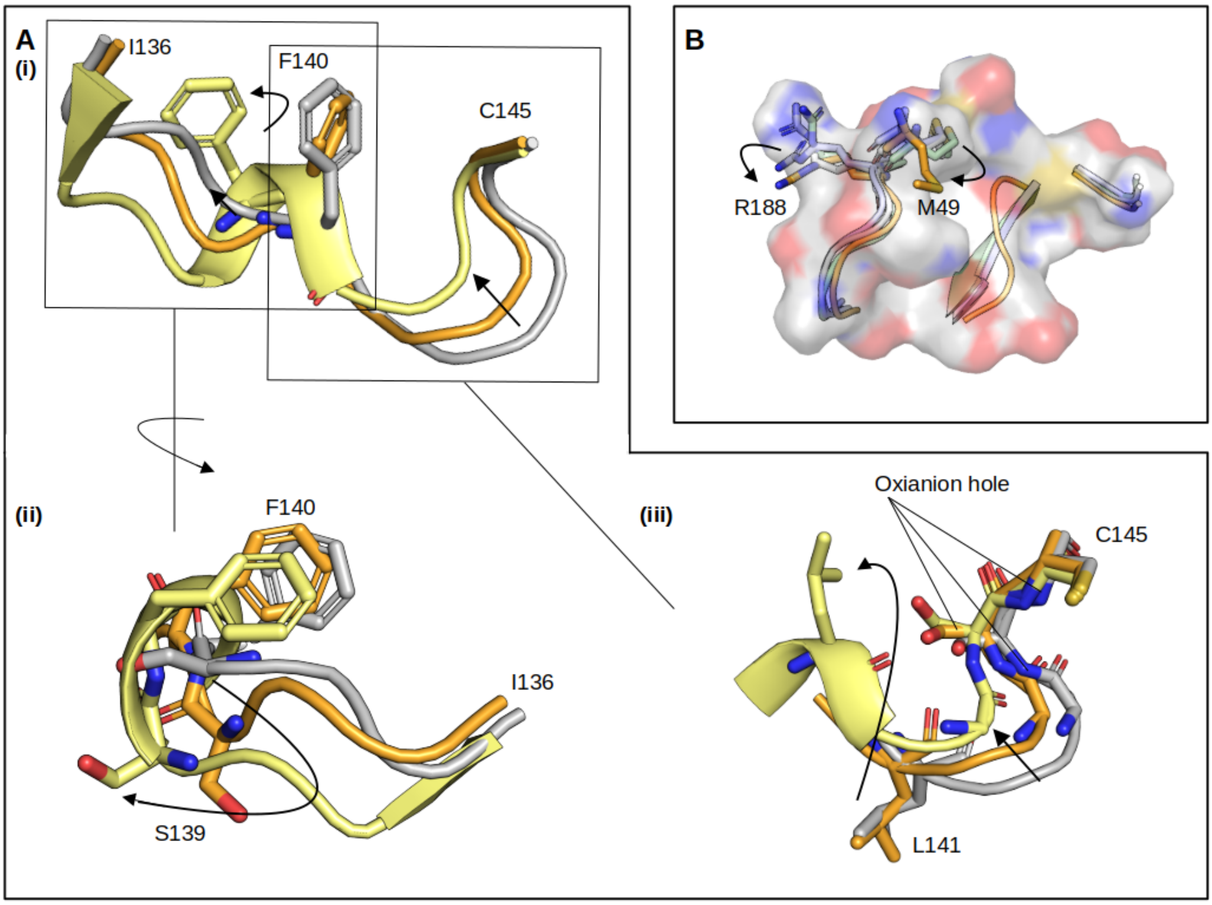


**Supplemental Figure 13. Comparison of the active site between M$^{pro}$ variants and the monomeric construct M$^{pro}$1-199.** Wild-type M$^{pro}$ (PDB: 7BB2) is depicted in grey, while the N214A variant (PDB: 9GI6) is orange and the monomeric M$^{pro}$1-199 construct (PDB: 7UJ9) in yellow (*12*, *13*). (**A**)(i) In the stabilized monomeric M$^{pro}$ structure, the oxyanion hole loop (I136 to C145) forms a $3_{10}$ helix (residues S139 to L141) not seen in the wild-type reference structure. While a clear $3_{10}$ helix was not found in the structure of the N214A mutant, we detected a loss in observable density and shift in mean position of residues L141 to G143, potentially reflecting an intermediate state between the monomer construct and wild type (see main text Fig. 5). (ii) Close-up view of the left side of panel (i), rotated for clarity. Amino acid S139 exhibits a side-chain flip coupled with realignment within the loop. (iii) Close-up view of the right side of the loop in panel (i), showing L141 occupying F140's original position to form a helix in the monomeric construct. (**B**) An alternative view of the M$^{pro}$ active site. Although the flexible residues R188 and M49 exhibit slight conformational shifts, the three different point mutations induce minimal changes to the active site architecture. Arrows highlight the changes that are observable, which are most dramatic in the N214A mutant.

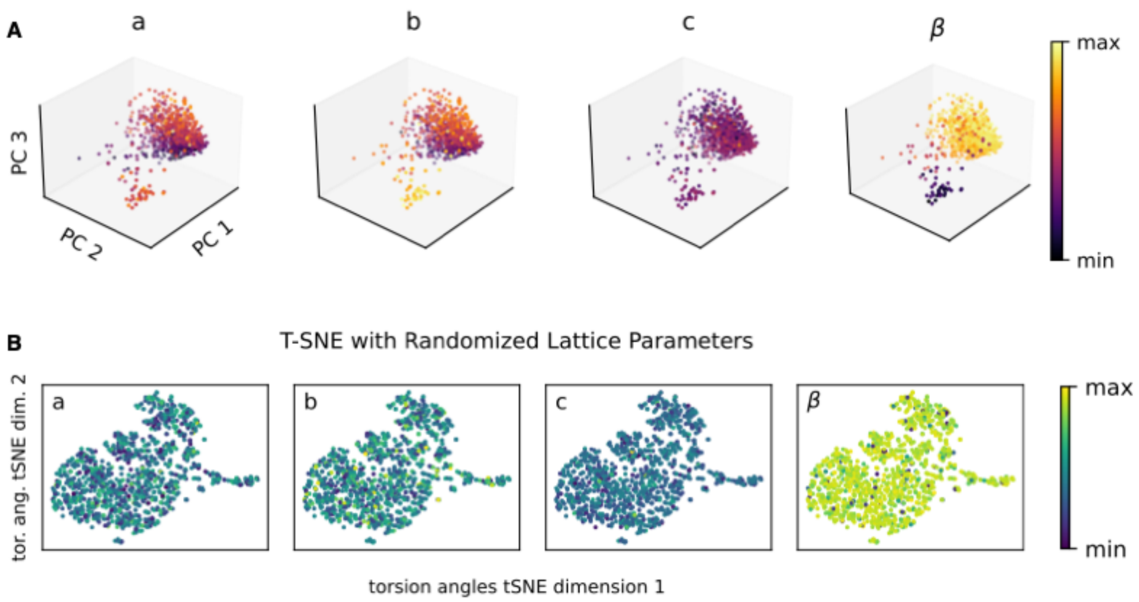


**Supplemental Figure 14. Controls for t-SNE analysis that support a link between protein structure and crystal lattice.** (**A**) PCA is presented as an alternative dimensionality reduction method to visualize the lattice-structure relationship. PCA was performed on the sine and cosine of each backbone dihedral angle, and three first (highest variance) principal components (PC1, PC2, PC3 respectively) were plotted. These components account for 19%, 8%, 6% of the total variance, respectively. The points are colored by the relative lattice parameter length, where the relevant lattice dimension is indicated above each plot. (**B**) The same t-SNE analysis as shown in main text Figure 6C, but with the lattice parameter values randomized. The lack of any structure is apparent.

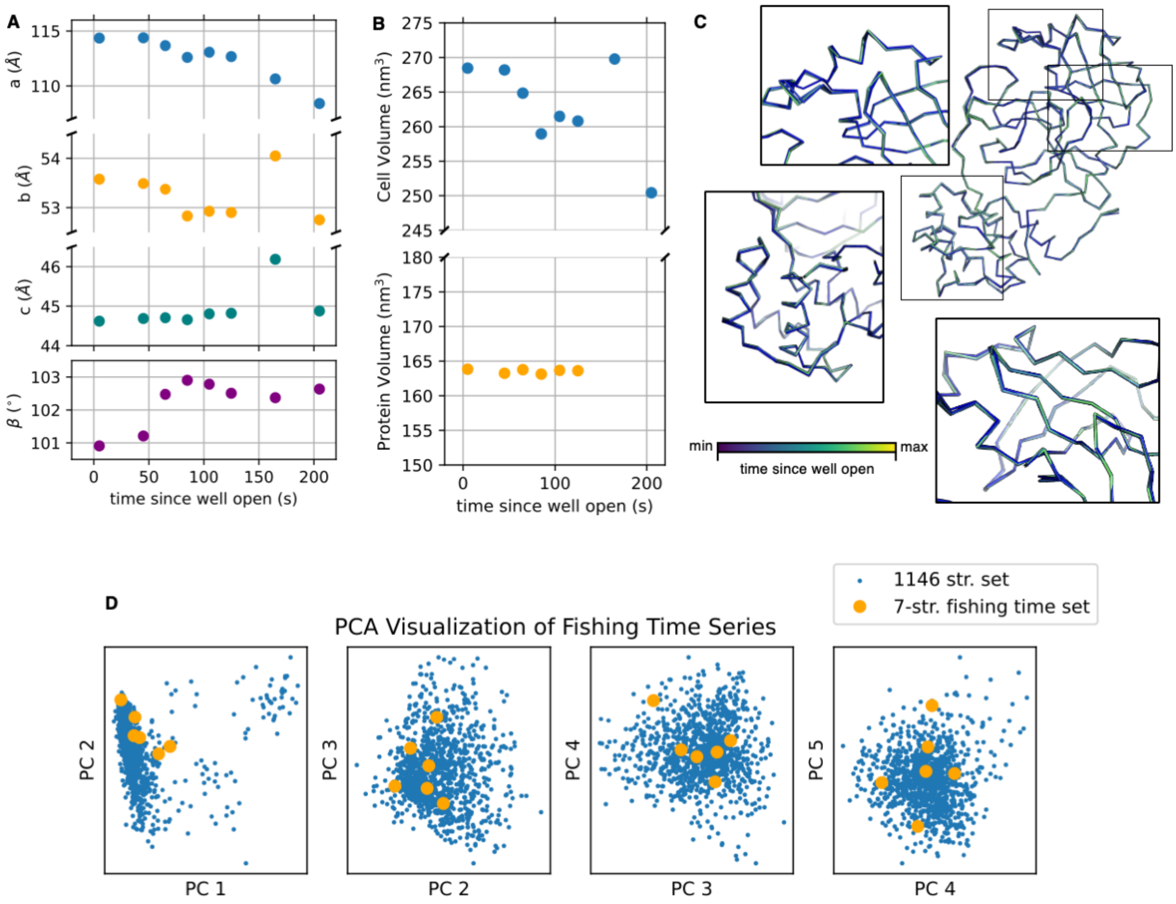


**Supplemental Figure 15. Delayed crystal fishing produces a continuous deformation of the M$^{pro}$ structure.** A single well of M$^{pro}$ crystals was opened and crystals were fished and frozen continuously. These crystals were then subjected to diffraction analysis and models were refined to the resulting data. (**A**) As a function of time since the well was opened, the cell parameters change continuously, (**B**) resulting in a contraction of the unit cell (top panel) but no appreciable change in the refined protein volume (bottom panel, protein volume determined by *3v* (*14*)). This implies that the change in cell volume is due to a loss of solvent as the crystals dry out. (**C**) The corresponding protein structures change continuously as a function of time since the well was opened. Shown are 6 structures from 5 seconds delay (blue) through 125 seconds delay (light green). Crystals fished at 165 and 205 seconds, the rightmost data points, did not produce sensible structures (*R*-factors >40%) and can be considered unreliable. The structural changes are similar to those seen when the set of M$^{pro}$ crystals analyzed in the main text is sorted by unit cell volume (Figure 6B). We conclude that hydration changes, caused for example by delays during crystal fishing, account for a major factor in generating the diversity of structures we analyzed. (**D**) As in Supplementary Fig. 13, the crystal structures from our large dataset were represented as dihedral angles and projected into a principal components space (PCA) and are shown as blue points. Then, the structures fished at specified time intervals were projected into the same space and are shown as orange points. The seven structures fished over time cover only a subset of the structural distribution observed in the larger 1146 structure dataset presented in the main text.

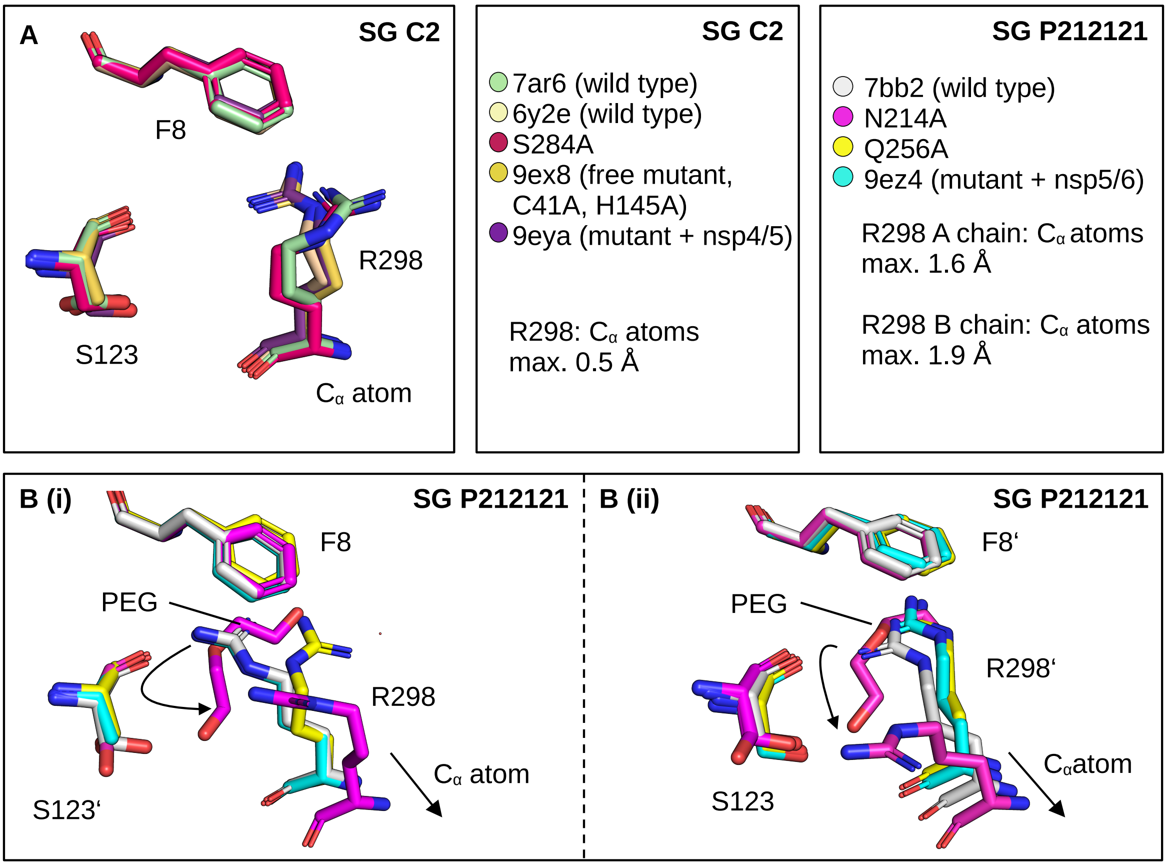


**Supplemental Figure 16**. **The backbone and sidechain positions of residue R298 depend on lattice packing**. R298 bridges the dimer interface, and its mutation to alanine has been shown to disrupt dimerization and correspondingly compromise enzyme turnover (*15*). As our covariance analysis did not identify this residue as a covariance hotspot, we inspected this residue's structure manually across a set of representative structures to understand why. (**A**) In the monoclinic C2 space group (structures with PDB IDs 6y2e, 7ar6, 9ex8, 9eya, and our S284A structure), the R298 sidechain generally displays weak electron density, often limited to the guanidine group, and its modeled position varies from structure to structure. For instance, in 7ar6, the sidechain is completely unresolved and displaced by DMSO, indicating high flexibility and a lack of stabilizing interactions. The $C_\alpha$ position of R298, however, is well-resolved and stable across these different structures. (**B**) In contrast, structures solved in the orthorhombic $P2_12_12_1$ space group (notably PDB IDs 7bb2 and 9ez4, as well as our Q256A and N214A structures) show consistently well-resolved and stable conformations of R298. For instance, in 7bb2, chain A exhibits full electron density for R298, stabilized by a π-stacking interaction with F8, while chain B retains guanidine density in the same orientation. Moreover, in this space group, the $C_\alpha$ position of R298 changes more significantly from structure to structure, by up to 1.9 Å. This comparison offers an explanation for why our covariance analysis, which reported $C_\alpha$ positions for a set of crystals in the C2 lattice, failed to identify R298 as a hotspot. In the space group we studied, the sidechain of R298 is mobile but the $C_\alpha$ atom is not, precluding a strong covariance signal. As this residue appears more mobile in the $P2_12_12_1$ space group, future studies would benefit from the study of multiple lattice packings and more powerful analysis methods that can capture sidechain motion and electron density strength directly.

## Supplemental References


1. I. J. Tickle, C. Flensburg, P. Keller, W. Paciorek, A. Sharff, C. Vonrhein, G. Bricogne, STARANISO. Global Phasing Ltd. [Preprint] (2018).
2. P. D. Adams, P. V. Afonine, G. Bunkóczi, V. B. Chen, I. W. Davis, N. Echols, J. J. Headd, L. W. Hung, G. J. Kapral, R. W. Grosse-Kunstleve, A. J. McCoy, N. W. Moriarty, R. Oeffner, R. J. Read, D. C. Richardson, J. S. Richardson, T. C. Terwilliger, P. H. Zwart, PHENIX: A comprehensive Python-based system for macromolecular structure solution. *Acta Crystallogr D Biol Crystallogr* **66**, 213–221 (2010).
3. B. Tom Burnley, P. V. Afonine, P. D. Adams, P. Gros, Modelling dynamics in protein crystal structures by ensemble refinement. *Elife* **2012** (2012).
4. W. Härdle, L. Simar, *Applied Multivariate Statistical Analysis: Second Edition* (2007).
5. S. P. Lloyd, Least Squares Quantization in PCM. *IEEE Trans Inf Theory* **28**, 129–137 (1982).
6. Molecular Dynamics Simulations Related to SARS-CoV-2, *D. E. Shaw Research* (2020)p. http://www.deshawresearch.com/resources_sarscov2.h.
7. T. S. Komatsu, Y. Koyama, N. Okimoto, G. Morimoto, Y. Ohono, M. Taiji, COVID-19 related trajectory data of 10 microseconds all atom molecular dynamics simulation of SARS-CoV-2 dimeric main protease. [Preprint] (2020).
8. T. Sztain, R. Amaro, J. A. McCammon, Elucidation of Cryptic and Allosteric Pockets within the SARS-CoV-2 Main Protease. *J Chem Inf Model* **61** (2021).
9. A. Douangamath, D. Fearon, P. Gehrtz, T. Krojer, P. Lukacik, C. D. Owen, E. Resnick, C. Strain-Damerell, A. Aimon, P. Ábrányi-Balogh, J. Brandão-Neto, A. Carbery, G. Davison, A. Dias, T. D. Downes, L. Dunnett, M. Fairhead, J. D. Firth, S. P. Jones, A. Keeley, G. M. Keserü, H. F. Klein, M. P. Martin, M. E. M. Noble, P. O'Brien, A. Powell, R. N. Reddi, R. Skyner, M. Snee, M. J. Waring, C. Wild, N. London, F. von Delft, M. A. Walsh, Crystallographic and electrophilic fragment screening of the SARS-CoV-2 main protease. *Nat Commun* **11**, 1–11 (2020).
10. S. Niebling, O. Burastero, J. Bürgi, C. Günther, L. A. Defelipe, S. Sander, E. Gattkowski, R. Anjanappa, M. Wilmanns, S. Springer, H. Tidow, M. García-Alai, FoldAffinity: binding affinities from nDSF experiments. *Sci Rep* **11** (2021).
11. Stephan Niebling, BioPhyPy. [Preprint] (2024).
12. A. Kovalevsky, A. Aniana, L. Coates, R. Ghirlando, N. T. Nashed, J. M. Louis, Visualizing the Active Site Oxyanion Loop Transition Upon Ensitrelvir Binding and Transient Dimerization of SARS-CoV-2 Main Protease. *J Mol Biol* **436** (2024).
13. N. T. Nashed, D. W. Kneller, L. Coates, R. Ghirlando, A. Aniana, A. Kovalevsky, J. M. Louis, Autoprocessing and oxyanion loop reorganization upon GC373 and nirmatrelvir binding of monomeric SARS-CoV-2 main protease catalytic domain. *Commun Biol* **5** (2022).
14. N. R. Voss, M. Gerstein, 3V: Cavity, channel and cleft volume calculator and extractor. *Nucleic Acids Res* **38**, 555–562 (2010).
15. E. Fornasier, S. Fabbian, H. Shehi, J. Enderle, B. Gatto, D. Volpin, B. Biondi, M. Bellanda, G. Giachin, A. Sosic, R. Battistutta, Allostery in homodimeric SARS-CoV-2 main protease. *Commun Biol* **7** (2024).
