# Supplementary material for: Statistical crystallography reveals an allosteric network in SARS-CoV-2 Mpro
Source: Commun Biol. 2026 May 2;9:602. doi: 10.1038/s42003-026-10127-w (PMC13135515; doi:10.1038/s42003-026-10127-w)
Supplement: Supplementary file 2 — Description of Additional Supplementary Files [file 42003_2026_10127_MOESM2_ESM.docx]

**Description of Additional Supplementary File**
File name: Supplementary data
Description: Source data have been uploaded in excel format, constituting five (5) total files:

- Raw SEC data (Supplementary Dataset 1),
- SEC concentration fits (Supplementary Dataset 2),
- raw ITC and NDF data (Supplementary Dataset 3),
- fit kinetics velocities (Supplementary Dataset 4) and
- Michaelis-Menten parameters (Supplementary Dataset 5)
